# The diabetic foot risks profile in Selebi Phikwe Government Hospital, Botswana

**DOI:** 10.4102/phcfm.v6i1.610

**Published:** 2014-10-17

**Authors:** Stephane Tshitenge, Adewale Ganiyu, Deogratias Mbuka, Joseph M. Shama

**Affiliations:** 1 Family physicians and lecturers, Department of family medicine, School of Medicine, University of Botswana, Botswana; 2 Hospital superintendent, Selebi Phikwe Government Hospital, Botswana

## Abstract

**Aim:**

The present study aimed: (1) to evaluate the proportion of each diabetic foot (DF) risk category, according to the International Working Group on the Diabetic Foot (IWGDF) consensus, in patients attending the diabetic clinic in Selebi Phikwe Government Hospital (SPGH) and (2) to examine some of the factors that may be associated with the progression to higher risk categories such as anthropometric measurements, blood pressure, glycosylated haemoglobin (HbA1c) and lipid profile.

**Methods:**

A retrospective, cross sectional chart review of patients who had attended the diabetic clinic in SPGH from January 2013 to December 2013 was performed. Patients were included if they had undergone a foot examination. Patients with amputation due to accident were excluded. The DF risk category was assessed by determining the proportion of patients in each of four risk categories, as described by the IWGDF consensus.

**Results:**

The study encompassed 144 records from patients reviewed for foot examination from January to December 2013. Patients’ ages were between 16 and 85 years, 46 (40%) were male and 98 (60%) were female. The majority (122, [85%]) of patients were in DF risk category 0, whilst a limited number of patients were classified in risk category 1 (10, [6.9%]), risk category 2 (7, [4.9%]) and risk category 3 (5, [3.5%]). Most of the patients had the type 2 diabetes mellitus (139, [97%; 95% CI 92% – 99%]). Patients’ ages were associated with the progressively higher DF risk categories. The adjusted odd ratio was 1.1 (95% CI 1.03-1.14; *p* = 0.004).

**Conclusion:**

The present study revealed that about 15% of patients attending the SPGH diabetic clinic were categorised in higher risk groups for diabetic foot; patients’ ages were linked to the higher DF risk categories.

## Abstrait

**Le profil des risques du pied diabétique à l'Hôpital gouvernemental de Selebi Phikwe, au Botswana**.

**Objectif:**

Cette étude a pour but: (1) d’évaluer la proportion de catégorie de risques du pied diabétique (DF), selon le Groupe de Travail International sur le document de consensus sur le pied diabétique (IWGDF), chez les patients fréquentant la clinique pour diabétiques de l'Hôpital gouvernemental de Selebi Phikwe (SPGH) et (2) d'examiner les facteurs associés à la progression vers des catégories de risques plus élevés tels que les mesures anthropométriques, la tension sanguine, l'hémoglobine glycosylée (HbA1c) et le taux de lipides.

**Méthodes:**

On a effectué une révision rétrospective et transversale des graphiques des patients qui avaient fréquenté la clinique pour diabétiques du SPGH de janvier 2013 à décembre 2013. Les patients qui avaient subi un examen du pied ont été acceptés. Les patients ayant subi une amputation à la suite d'un accident ont étés exclus. La catégorie à risque du pied diabétique a été évaluée par la détermination de la proportion de patients dans chacune des quatre catégories à risque, comme il est décrit dans le document de consensus IWGDF.

**Résultats:**

L’étude comprend 144 dossiers de patients évalués pour l'examen du pied de janvier à décembre 2013. Les patients avaient de 16 à 85 ans, 46 (40%) étaient des hommes et 98 (60%) des femmes. La majorité (122, [85%]) des patients étaient dans la catégorie à risque de pied diabétique 0, alors qu'un nombre limité de patients étaient classés dans la catégorie à risque 1 (10, [6.9%]), la catégorie à risque 2 (7, [4.9%]) et la catégorie à risque 3 (5, [3.5%]). La majorité des patients avaient le type 2 de diabète mellitus (139, [97%; 95% CI 92% - 99%]). L’âge des patients correspondait à des catégories à risque progressivement plus élevé de diabète du pied. Le rapport de risques ajusté était de 1.1 (95% CI 1.03-1.14; *p* = 0.004).

**Conclusion:**

Cette étude a révélé qu'environ 15% des patients fréquentant la clinique pour diabétiques de SPGH étaient classés dans des groupes à risque plus élevé de pied diabétique; l’âge des patients était lié aux catégories de risques plus élevé de pied diabétique.

## Introduction

Lower limb problems such as foot ulceration, infection and amputation are common in people with diabetes. ^1, 2, 3, 4, 5^ Recent reports have highlighted the significance of prompt recognition of the high-risk foot and the standardised provision of preventive measures, ^6, 7^ as they can help to avoid the development of foot lesions, minimise morbidity and costs resulting from those complications. Strategies such as patient and staff education, multi-disciplinary management of foot ulcers, and close monitoring can lessen the amputation rate.^8, 9, 10, 11^

High risk of foot complications is associated with: a history of prior ulcer or amputation, Charcot foot, poor glycaemic control, trauma, peripheral neuropathy and/or peripheral vascular diseases, infections, foot structure deformity, impaired vision, old age, male gender, and ethnicity (black people and Hispanic people).^4, 12, 13, 14^

Several risk-stratification schemes have been suggested; none of these classification systems has been universally adopted to foretell complications. The International Working Group on the Diabetic Foot (IWGDF) system seems to be the most endorsed and simplified risk-stratification system used.^15^ This system classifies patients into four risk categories. In risk category 0, patients are considered to have protective sensation or there is no foot deformity. In risk category 1, patients are considered to have an impending risk, as they have a loss of protective sensation (LOPS). In risk category 2, patients have high risk, as they have LOPS in their feet with evidence of the high-pressure zone (callus/deformity) or poor circulation. In risk category 3, patients have a history of plantar ulceration or neuropathic fracture (Charcot foot). ^4, 12, 15^ Risk stratification also serves as a guide to schedule patient review; risk category 0, 1, 2 and 3 patients are reviewed annually, semi-annually, quarterly and monthly to quarterly, respectively.^4^

The aim of the present study was: (1) to evaluate the proportion of each diabetic foot (DF) risk category, according to IWGDF consensus,^4^ in patients attending a diabetic clinic in Selebi Phikwe Government Hospital (SPGH), a district primary care facility in Botswana; and (2) to examine some of the factors that may be associated with the progression to higher risk categories such as anthropometric measurements, blood pressure, glycosylated haemoglobin (HbA1c) and lipid profile.

### Contribution to field

There is a paucity of data available from Sub-Saharan Africa on DF risk categories and the predisposing factors. The majority of available data are from studies conducted in secondary and tertiary settings. Therefore, it was deemed necessary to conduct such a study in a primary care setting. It is hoped that the public will benefit from this study, as it attempts to document the prevalence of DF risk categories in primary healthcare in Selebi Phikwe, Botswana and the Sub-Saharan region.

### Ethical considerations

Ethical clearance for the study was obtained from the University of Botswana (URB/IRB/1406) and the Ministry of Health [PPME 13/18/1 VIII (79)]. Permission for data collection at SPGH was also obtained from the hospital management. A waiver of patient consent was obtained from the Institutional Review Board (University of Botswana) since the study required only examination of routinely maintained medical records.

## Research methods and design

### Study design

A retrospective, cross sectional chart review was performed of patients who had attended the diabetic clinic in SPGH from January 2013 to December 2013.

### Context and sampling of the study

The present study involved reviewing all diabetes mellitus (DM) patients’ records: both type 1 and type 2 diabetes mellitus. Patients were booked for foot assessment as they arrived at the clinic and they were reviewed as per IWGDF recommendations.^4^

The SPGH is situated in the Central District of Botswana. It provides primary healthcare in outpatient clinics such as the diabetic clinic. The clinic has been operational since October 2010; in 2011, one nurse had formal training on diabetic care.

### Data collection and procedure

Records of patients booked for foot review during the study period were selected. Those from patients with amputation due to accident were excluded.

Data were collected from a checklist form used routinely in the clinic, which captures information such as: demographics (gender, age); type of DM; history of hypertension; and anthropometric measurements (weight, height, body mass index, waist circumference). Laboratory data (blood sugar, HbA1c, kidney function, lipid profile) and clinical variables such as a DF risk category were collected as well. The laboratory results (HbA1c, lipid profile) obtained within the previous six months were used for the study.

### Data analysis

Data distribution was checked, then summarised by calculating the mean ± standard deviation (s.d.) for normally distributed variables, and the frequency in percentages for binomial and the median ± interquartile range (IQR), if skewed. A Kruskal-Wallis rank test was performed to measure the differences between the medians of factor DF risk category with regards to independent variables such as: age, hypertension, systolic and diastolic blood pressure, body mass index (BMI), blood sugar level, HbA1c, LDL-C, HDL-C, total cholesterol and triglyceride. The Tukey's quick test was used as a *post hoc* test to find where these differences between median DF risk categories lied. To control for possible confounding variables, variables were selected with a significant level of association (*p* ≤ 0.2), their normality, linearity and homoscedasticity were checked and they were fitted into the ordinal regression model. Results were expressed as a coefficient, adjusted odds ratio (OR) and 95% confidence interval (CI). All statistical analyses were carried out using an R software version 3.0.0 with R commander package version 1.9-6. The level of significance was set at *p* < 0.05.

## Results

In 2013, the SPGH clinic registered 402 patients. Of these, 149 patients were included in the present study because they were reviewed for foot examination from January to December 2013. Five records were discarded, as they had missing data on most sections. There were no patients reviewed with an amputation during the study period.

### Demographic characteristics of the study population

Out of the 144 records included in the present study, 46 (40%) were male and 98 (60%) were female. Patients were aged between 16 and 85 years; their mean age was 55 years old (95% CI 53-57 years old).

### Diabetic foot risk category

The majority (122, [85.0%]) of patients in the present study were in the DF risk category 0; whilst fewer were classified in risk category 1 (10, [6.9%]), risk category 2 (7, [4.9%]) and risk category 3 (5, [3.5%]). Overall, the proportion of patients with LOPS, high-pressure zone (callus and/or deformity), poor circulation, history of plantar ulceration or neuropathic fracture deformity was 15.0%.

### Clinical and laboratory profile of the study population

As shown in Table 1, most of the patients in the study had type 2 DM (139 [97%; 95% CI 92% - 99%]). About three-quarters (74%; 95% CI 64% - 82%) of patients were diagnosed with hypertension. The average systolic blood pressure was 140 mm Hg (95% CI 135 mmHg - 144 mmHg). The majority (62/105, [59%]) had uncontrolled blood sugar (HbA1c above 7.0%), the mean HbA1c was 8.1% (95% CI 7.7% - 8.7%). The mean low-density lipoprotein cholesterol was 3.6 mmol/L (95% CI 2.9 mmol/L - 4.3 mmol/L). More than half of the patients had a BMI above 28 kg/m^2^ (95% CI 26 kg/m^2^ - 40 kg/m^2^).

**TABLE 1:** Clinical and biological profile of patients reviewed for foot examination at the diabetic clinic, January to December 2013.

Profile	Variables						95% CI

	%	*n*	mean	s.d.	Median	IQR	
Type 2 diabetes mellitus	97	139	-	-	-	-	96-98
Hypertension number	70	101	-	-	-	-	65-75
Systolic blood pressure (mmHg)	-	-	140	27	-	-	135-144
Diastolic blood pressure (mmHg)	-	-	77	11	-	-	75-78
Body mass index (kg/m^2^)	-	-	-	-	28	8.2	26-40
Waist circumference (cm)	-	-	106	13	-	-	102-111
Fasting blood sugar (mmol/L)	-	-	8.0	3.5	-	-	7.4-8.5
Haemoglobin A1c (%)	-	-	8.1	2.3	-	-	7.7-8.7
High density lipoprotein cholesterol (mmol/L)	-	-	1.2	0.4	-	-	1.1-1.3
Low-density lipoprotein cholesterol (mmol/L)	-	-	3.6	0.9	-	-	2.9-4.3
Triglyceride (mmol/L)	-	-	-	-	1.6	1.0	1.4 -1.8
Total cholesterol (mmol/L)	-	-	5.1	1.1	-	-	4.9-5.3

SPGH, Selebi Phikwe Government Hospital; s.d., standard deviation; IQR, interquartile range; mmol/L, millimol per litre.

### Association between diabetic foot risk category with demographic and clinical variables

**FIGURE 1:**
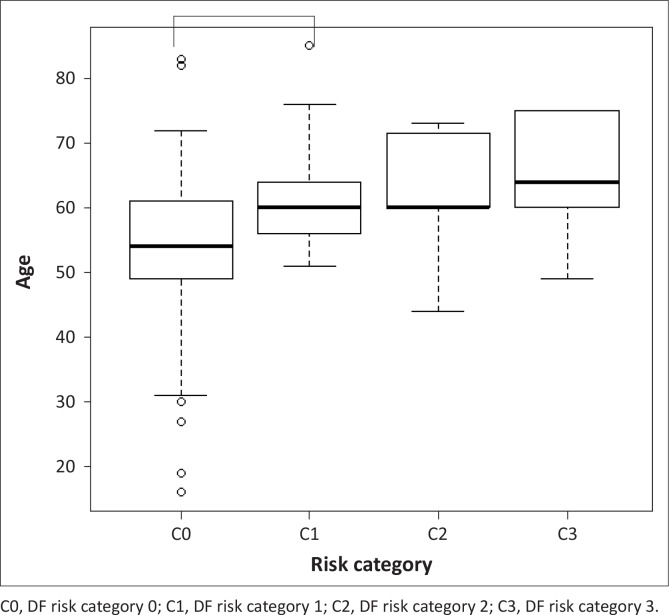
Boxplots of the age for each of the DF risk category in patients reviewed for foot examination at the diabetic clinic, January to December 2013.

The only demographic variable that was significantly different between risk categories was age (*p* = 0.005, as shown in Table 2). Subsequent analysis (depicted in Figure 1) shows that median age is significantly different only between categories 0 and 1 (*p* < 0.001). The results of the ordinal logistic regression analysis are shown in Table 3. Patients’ ages were associated with the progressively higher DF risk categories. The adjusted odd ratio was 1.1 (95% CI 1.03 - 1.14; *p* = 0.004).

**TABLE 2:** Kruskal-Wallis test, association between diabetic foot risk category and variables in patients reviewed for foot examination at the diabetic clinic, January to December 2013.

Variables	Diabetic foot risk category				Kruskal-Wallis *p-*value

	0	1	2	3	
Age, mean (s.d.), year	54.0 ± 9.9	60.0 ± 8.6	60.0 ± 1.4	64.0 ± 7.7	0.005
Systolic blood pressure, mean (s.d.), mmHg	134.0 ± 25.2	137.0 ± 33.1	138.0 ± 20.5	167.0 ± 20.1	0.13
Diastolic blood pressure, mean (s.d.), mmHg	76.0 ± 11.5	76.0 ± 9.4	79.0 ± 1.4	79.0 ± 4.7	0.72
Body mass index, median (IQR), kg/m^2^	30	27	27	24	0.25
Waist circumference mean (s.d.), cm	104.0 ± 13.0	108.0 ± 15.0	108.0	97.0	0.36
Fasting blood sugar, mean (s.d.), mmol/L	7.3 ± 3.6	7.9 ± 2.1	7.3 ± 0.6	7.3 ± 2.4	0.8
Haemoglobin A1c, mean (s.d.), g%	7.7 ± 2.3	6.9 ± 3.8	6.7	7.7 ± 0.96	0.84
High density lipoprotein cholesterol, mean (s.d.), mmol/L	1.1 ± 0.4	1.1 ± 0.3	1.2	1.0 ± 0.25	0.5
Low density lipoprotein cholesterol, median (IQR), mmol/L	3.4 ± 0.99	3.3 ± 0.4	3.2	3.3	0.98
Triglyceride, median (IQR), mmol/L	1.6 ± 1.04	1.9 ± 0.8	1.4	1.5 ± 1.1	0.37
Total cholesterol, median (SD), mmol/L	5.2 ± 1.2	5.1 ± 0.8	5.0	4.8 ± 0.6	0.48

SPGH, Selebi Phikwe Government Hospital; s.d., standard deviation; IQR, interquartile range; mmol/L, millimol per litre; kg/m2, kilogram per meter square. *p* < 0.001

**TABLE 3:** Ordinal logistic regression with the DF risk category as the dependent variable and both age and SBP as independent variables, in patients reviewed for foot examination at the diabetic clinic, January to December 2013.

Risk factors	Regression coefficient	Two tailed *p*-value	Adjusted odd ratio	95% CI
Age	0.08	0.004	1.1	1.03-1.14
SBP	0.02	0.1	1.0	0.99-1.04

SPGH, Selebi Phikwe Government Hospital; DF, diabetic foot; SBP, systolic blood pressure.

The study did not detect any significant differences between the median of DF risk categories in other variables, as their *p* values were above 0.05.

## Discussion

The present study revealed that amongst the patients reviewed for foot examination in the SPGH diabetic clinic, the majority (85%) were in DF risk category 0, whilst 15% were categorised in higher risk groups. A total of 6.9% of patients were classified in risk category 1, 4.9% in risk category 2 and 3.5% in risk category 3. One primary care setting study produced comparable results of prevalence with 8.4% for risk category 1, and 4.5% for risk category 2; but the prevalence of risk category 3 was higher (13.0%).^16^ In another study from a tertiary care setting, a higher prevalence of 17% for risk category 1, 11% for risk category 2 and 7% for category 3 was reported.^17^ The present study's patients were in lower-risk categories than from the previous studies. It can be speculated that there was misclassification of the patients’ DF to a lower-risk group in the present study; this could be due to only one nurse having had formal training for diabetic foot care. On the other hand, the present study's findings may reflect the true prevalence; if so, there would be a need to elucidate with further study factors that contributed to the lower DF risk classification of the present study's population.

In the present study, the majority of patients had hypertension, were overweight or obese (70%); 59% had uncontrolled blood sugar for at least the past three months (mean HbA1c = 8.1%) and showed elevated levels of LDL-C (mean LDL-C = 3.6 mmol/L). The partnership between hypertension and obesity is a common finding in type 2 diabetes. Biologic parameters such as HbA1c and LDL-C are not desirable for diabetic patients ^18, 19, 20^; thus, they did not show an association with higher DF risk categories. These findings showed similarity with a recent study.^17^ The patients in the present study had a mean age of 55 years, and it is known that type 2 diabetes patients over the age of 50 years struggle to regulate their blood sugar.^21^

The present study's findings revealed that patient's ages showed significant differences in the medians between the DF risk categories; this was trivial only between DF risk categories 0 and 1 (*p* < 0.001). These findings were comparable with previous reports.^22^ The present study's findings showed dissimilarity with a central Saudi Arabian study, as it failed to establish a positive association between increased SBP with the progressively higher DF risk categories.^23^ It was not part of the present study to assess the length of time that a patient had diabetes and its influence to the progression to higher DF risk categories.

There is little known about DF risk-category prevalence in primary healthcare settings in Sub-Sahara Africa. The importance of screening and sorting DF risk categories has the merit of being able to recognise patients in danger of lower extremity events and to implement time-appropriate care, more especially amongst older patients.

The limitations of the present study were: its retrospective and cross-sectional design, which consequently meant that the causal relationships between DF risk categories and variables, such as age, could not be recognised. Further investigations with a different design are recommended in order to clarify the influence of misclassification of DF risk categorisation.

### Recommendations

It is recommend that foot examination and risk categorisation should become part of the routine management of diabetic patients in primary care in Botswana and Sub-Saharan Africa. More healthcare providers should be empowered with these skills. A more rigorous, prospective and multi-centre study is required to shed more light on the magnitude of the DF risk in Botswana.

### Conclusion

The present study revealed that about 15% of patients attending the SPGH diabetic clinic were categorised in the high-risk groups for diabetic foot; patients’ ages were linked to the higher DF risk category. The present study provides additional data on the prevalence of DF risk categories in primary care settings in Botswana and the Sub-Saharan African region. Further prospective, large and multi-centre investigations are required to endorse these findings.
